# A custom GPT-based model for the automated analysis and interpretation of antimicrobial susceptibility tests in Gram-negative bacteria

**DOI:** 10.1093/jacamr/dlag132

**Published:** 2026-07-27

**Authors:** John Sprockel Díaz, Sharon Sotomonte, Alberto Buitrago

**Affiliations:** Research Institute and Artificial Intelligence Lab – “ProfundaMente” – Universidad FUCS, Internal Medicine Department – Hospital de San José de Bogotá, Bogotá, Colombia; Internal Medicine Program – Universidad FUCS, Bogotá, Colombia; Faculty of Medicine – Universidad FUCS, Head of the Infectious Diseases Service – Hospital de San José de Bogotá, Bogotá, Colombia

## Abstract

**Introduction:**

Antimicrobial resistance in Gram-negative bacilli represents a growing clinical challenge that demands innovative tools to optimize antibiogram interpretation and support safe therapeutic decisions.

**Objective:**

To develop and validate a custom generative pre-trained transformer (GPT) model for antibiogram interpretation, comparing its performance with an alternative model based on Gemini (Gems).

**Methods:**

A retrospective, cross-sectional diagnostic accuracy study was conducted following the STARD-artificial intelligence (AI) guideline. A total of 210 antibiograms were analysed, balanced across controls and five resistance mechanisms (penicillinases, broad-spectrum β-lactamases, extended-spectrum β-lactamases, AmpC and Carba-R). The custom GPT, configured through prompt engineering and retrieval-augmented generation, was evaluated against expert interpretation and compared with Gems. Performance metrics (sensitivity, specificity, precision, *F*1-score, receiver operating characteristic/precision–recall area under the curve and Cohen’s Kappa) were calculated, and errors were classified by clinical impact as overtreatment or undertreatment.

**Results:**

The GPT achieved an accuracy of 95.7% (95% confidence interval: 92.0–98.0) and a Kappa of 0.948, outperforming Gems (91.9% and 0.902, respectively). Both models showed good performance, but GPT displayed greater consistency across metrics, with sensitivities and *F*1-scores >90% in all classes. Clinically relevant errors were fewer and less severe with GPT (9 versus 17 in Gems), with markedly lower spectrum penalty and treatment failure risk indices.

**Conclusions:**

This study provides proof-of-concept evidence that a custom GPT model can accurately interpret antibiograms of Gram-negative bacilli under controlled retrospective condition. Although these findings are promising, external multicentre validation, expansion to other pathogens and resistance mechanisms and further assessment as a clinical decision-support tool are required before routine clinical implementation.

## Introduction

Antimicrobial resistance (AMR) has emerged as one of the greatest threats to global public health. The sustained rise in infections caused by multidrug-resistant bacteria has generated an unprecedented health and socioeconomic crisis, with projections estimating up to 10 million deaths annually by 2050, surpassing those caused by cancer and diabetes combined.^1,2^ This phenomenon affects not only hospital morbidity and mortality but also imposes a substantial economic burden on healthcare systems by prolonging hospital stays, increasing the need for last-line therapies,^3,4^ and intensifying the selective pressure that drives the emergence of new resistance mechanisms.^5^

In this context, antimicrobial susceptibility testing (AST) and antibiograms remain essential tools for guiding the rational use of antibiotics in bacterial infections.^6^ However, conventional methods present significant limitations: results typically require 24–72 h, or even longer for slow-growing organisms, and rely on manual interpretation, which introduces considerable inter-observer variability.^7,8^ This delay often forces the initiation of empirical therapies that may be ineffective if the pathogen is resistant or unnecessarily broad, thereby contributing to the ongoing expansion of AMR.^9^

To address these challenges, digital microbiology and diagnostic automation technologies have emerged as promising alternatives.^10,11^ Among these innovations, large language models (LLMs), such as the generative pre-trained transformer (GPT) family, represent a major technological leap. Their ability to process natural language and reason over clinical data enables automated interpretation of antibiograms, improved detection of resistance mechanisms and rapid generation of structured clinical reports using standardized prompts and predefined output formats. Furthermore, LLM-based clinical chatbots can assist with specimen collection, interpretation of complex results and communication with healthcare personnel, thereby enhancing laboratory efficiency and supporting evidence-based therapeutic decisions.^12^

Custom GPTs, introduced in November 2023, are configurable versions of ChatGPT that allow users to tailor models for specific tasks or domains by combining customized instructions, uploaded knowledge and integrated capabilities.^13^ Similarly, other platforms, such as Google Gemini’s ‘Gems’, have developed comparable tools that offer flexible personalization and adaptation to user needs.

The present study aimed to develop and evaluate, as a proof of concept, a custom GPT model for the automated interpretation of antibiograms, designed to infer distinct bacterial resistance mechanisms and generate structured clinical reports. The model was conceived as a potential clinical decision-support tool, rather than as a system for autonomous diagnosis or immediate integration into routine laboratory workflow.

## Methods

### Development of the custom GPT

The model was configured through a structured prompt-engineering approach, using a master prompt to define the model’s role, task scope, reference knowledge, response structure and safety constraints. This configuration did not involve additional training or fine-tuning of the underlying LLM. For contextual grounding, a retrieval-augmented Generation (RAG) framework was implemented, integrating specialized literature, including as a core reference the article ‘Mechanisms of Antibiotic Resistance in Important Gram-Positive and Gram-Negative Pathogens and Novel Antibiotic Solutions’.^14^ The models received the antibiogram results as categorical susceptibility interpretations (S/I/R) as reported by the laboratory.

Subsequently, iterative prompt refinement was conducted using pilot tests with five representative cases for each resistance mechanism. These tests allowed for iterative adjustments to the prompt to optimize phenotypic identification and improve the clarity, coherence and safety of the generated clinical reports. The finalized prompt, designed for phenotypic interpretation of antibiograms and identification of β-lactam resistance mechanisms, including β-lactamase-mediated and non-β-lactamase-mediated mechanisms, was deployed as a custom GPT within the ChatGPT platform (GPT-5 model) and as a Gem within the Google Gemini platform (Gemini 2.5 Pro model). The prompt used to configure the custom GPT is provided as Supplementary material (available as Supplementary data at *JAC-AMR* Online).

### Study design

A retrospective, cross-sectional diagnostic accuracy study was conducted following the Standards for Reporting of Diagnostic Accuracy-artificial intelligence (STARD - AI) guideline for studies focused on AI.^15^

### Sample selection

Antibiogram reports generated by the Microbiology Laboratory of the Sociedad de Cirugía de Bogotá—Hospital de San José between 1 November 2024 and 31 May 2025 were included. Eligible reports were identified from the laboratory records and reviewed sequentially by the study team to determine whether they met the predefined inclusion criteria. Cases corresponded to fermenting and non-fermenting Gram-negative bacilli and were selected by convenience sampling to ensure balanced representation across six phenotypic groups: non-resistant controls, penicillinases, broad-spectrum β-lactamases (functional Group 2b, BSBL), extended-spectrum β-lactamases (functional Group 2be, ESBL), AmpC (overexpression or plasmid-mediated) and carbapenem resistance (Carba-R).

Inclusion criteria required reports validated by an infectious disease specialist and antibiotic panels containing at least 12 first-line agents and one carbapenem. Duplicates (≤7 days, same patient and organism) and incomplete records were excluded. Anonymization was performed after case eligibility and phenotypic classification had been established before the reports were entered into the GPT and Gems platforms. The process consisted of removing all direct patient identifiers, including names, identification numbers, medical record numbers, dates of birth, admission numbers and any other administrative identifiers. In addition, clinical or diagnostic information not required for antibiogram interpretation was removed to avoid explicit diagnostic clues. Identifiable data were initially handled only by the investigator responsible for database construction and were stored locally on that investigator’s password-protected personal computer, rather than on an institutional secure server. The identifiable dataset was not shared with the other members of the research team. After anonymization, only the de-identified study database was used for model evaluation and statistical analysis.

Only the bacterial identification and categorical antimicrobial susceptibility results (S/I/R) were retained for model analysis. Each case was assigned a study code, and no identifiable patient information was provided to the models. Chat history was deleted after each session, memory was disabled, and the ChatGPT and Gemini account settings specified that submitted data were not used for training future models.

### Reference standard

Model interpretations were compared against expert readings performed by an experienced clinical infectious disease specialist. Immunochromatographic confirmation using CARBA 5^®^ was available for 12 of the 33 Carba-R isolates (36.4%) and was not systematically performed in all cases. Accordingly, this category was reported as carbapenem resistance (Carba-R), rather than carbapenemase production.

### Statistical analysis

For each included case, the reference phenotypic category, the GPT classification and the Gems classification were entered into a structured database and compared using the reference standard to calculate diagnostic performance metrics. The distribution of cases across resistance categories was evaluated, comparing correct and incorrect classifications for each model. Subsequently, the overall diagnostic accuracy of GPT and Gems was estimated with 95% confidence intervals (95% CIs) using the exact binomial method. Cohen’s Kappa coefficient with bootstrap-derived CIs was calculated to assess agreement with the reference standard. Additionally, class-specific performance metrics (sensitivity, specificity, precision and *F*1-score) were analysed with CIs computed using the Wilson method, in order to capture variability in performance across resistance mechanisms.

The discriminative ability of the models was further explored through receiver operating characteristic (ROC) and precision–recall (PR) curves using a one-versus-rest approach, calculating the area under the curve (AUC) for each class with 95% CIs estimated by bootstrap resampling.

To formally compare both models, a *z*-test for difference of proportions was applied to assess discrepancies in overall accuracy, a paired bootstrap was used for differences in Kappa and a non-parametric bootstrap was employed to evaluate differences in *F*1-score by class and on average. In addition, tests for difference of proportions were used to contrast sensitivity, specificity and precision between GPT and Gems for each resistance mechanism.

A separate analysis of clinically relevant errors was performed based on the confusion matrix. Each error was categorized as overtreatment (unnecessary escalation of antibiotic spectrum) or undertreatment (risk of therapeutic failure), and weighted using a scale proportional to the magnitude of the discrepancy. From these data, two composite indices were calculated: the Spectrum Penalty Index and the Failure Risk Index, designed to approximate the potential clinical impact of model errors on therapeutic decision-making (see Supplementary material).

### Sample size calculation

Sample size was determined assuming an expected sensitivity of 0.90 (95% CI, margin of error ±0.07), requiring approximately 25 isolates per resistance mechanism. With five categories and one control group, a total range of 80–120 antibiograms was established, selected through stratified convenience sampling to ensure 10–25 cases per mechanism and 20–60 controls.

All analyses were conducted in Python 3.11 using the libraries pandas, scikit-learn, statsmodels, scipy and pingouin. Statistical significance was set at *P* < 0.05.

## Results

A total of 210 antibiograms were analysed, exceeding the initially estimated minimum sample size and thereby strengthening the precision of the performance estimates. Non-fermenting Gram-negative bacilli accounted for 20 cases, including *Pseudomonas aeruginosa* (*n* = 9), *Acinetobacter baumannii* (*n* = 6), *Acinetobacter lwoffii* (*n* = 3), *Alcaligenes faecalis* (*n* = 1), and *Burkholderia cepacia* (*n* = 1). Cases were distributed across six phenotypic categories: non-resistant controls (*n* = 55; 26.2%), penicillinases (*n* = 37; 17.6%), BSBL (*n* = 19; 9.0%), ESBL (*n* = 31; 14.8%), AmpC β-lactamases (*n* = 35; 16.7%) and Carba-R (*n* = 33; 15.7%). The balanced distribution of isolates enabled a robust comparative analysis across resistance mechanisms.

When comparing both models, GPT outperformed Gems, achieving a diagnostic accuracy of 95.7% (95% CI: 92.0–98.0) versus 91.9% (95% CI: 87.4–95.0), and a Cohen’s Kappa of 0.948 (95% CI: 0.912–0.982) compared to 0.902 (95% CI: 0.860–0.940). Although both reached a level of agreement classified as almost perfect, the difference in Kappa was statistically significant by bootstrap analysis (Δ = 0.046; 95% CI: 0.006–0.094; *P* = 0.02), confirming greater consistency of the GPT model.

**Figure 1.**
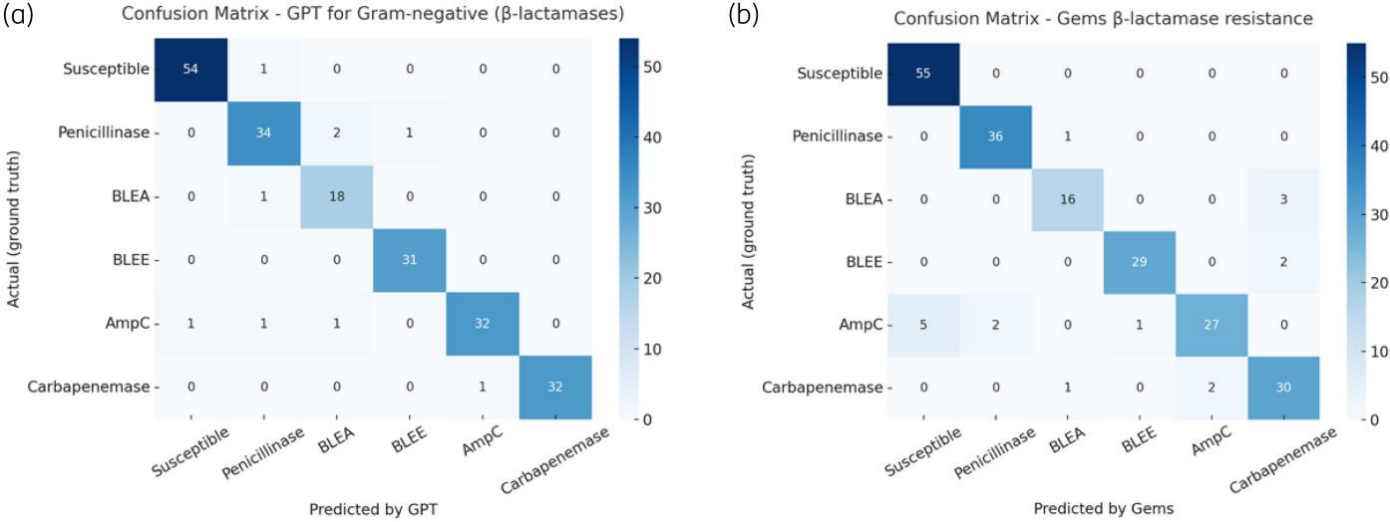
Heatmap of the confusion matrices for classifications obtained by the two models: (a) GPT and (b) Gems.

The McNemar test indicated that in both models, errors were not randomly distributed but instead clustered around specific pairs of resistance mechanisms (*P* < 0.05). The confusion matrices (Figure 1) confirmed the overall strong performance of both systems. GPT exhibited only a few misclassifications, limited to confusions between phenotypically related mechanisms, whereas Gems showed a higher number of errors, particularly in AmpC and BSBL, including occasional misclassifications as susceptible isolates or Carba-R producers.

Table 1 shows that both models achieved high overall performance values; however, GPT demonstrated a more consistent behaviour, with sensitivities and *F*1-scores exceeding 90% across all categories. In contrast, Gems exhibited performance drops in BSBL and, more notably, in AmpC, which accounts for its lower overall concordance. Both models, however, showed excellent performance for ESBL and Carba-R categories, with results approaching 100%.

**Table 1.** Performance of both models in the classification of resistance mechanisms by class

Class	Sensitivity GPT	Sensitivity Gems	*P* (Sens)	Precision GPT	Precision Gems	*P* (Prec)	*F*1 GPT	*F*1 Gems	*P* (*F*1)	Specificity GPT	Specificity Gems	*P* (Spec)
Susceptible	0.982	1.000	0.315	0.982	0.917	0.117	0.982	0.957	0.230	0.994	0.968	0.099
Penicillinase	0.919	0.973	0.304	0.919	0.947	0.621	0.919	0.960	0.240	0.983	0.988	0.652
BSBL	0.947	0.842	0.290	0.857	0.889	0.768	0.900	0.865	0.634	0.984	0.990	0.653
ESBL	1.000	0.935	0.151	0.969	0.963	0.963	0.984	0.951	0.236	0.994	0.994	1.000
AmpC	0.914	0.771	0.101	0.970	0.931	0.479	0.941	0.844	0.016	0.994	0.989	0.562
Carba-R	0.970	0.909	0.302	1.000	0.857	0.026	0.985	0.882	<0.001	1.000	0.972	0.024

BSBL, broad-spectrum β-lactamases; ESBL, extended-spectrum β-lactamases.

**Figure 2.**
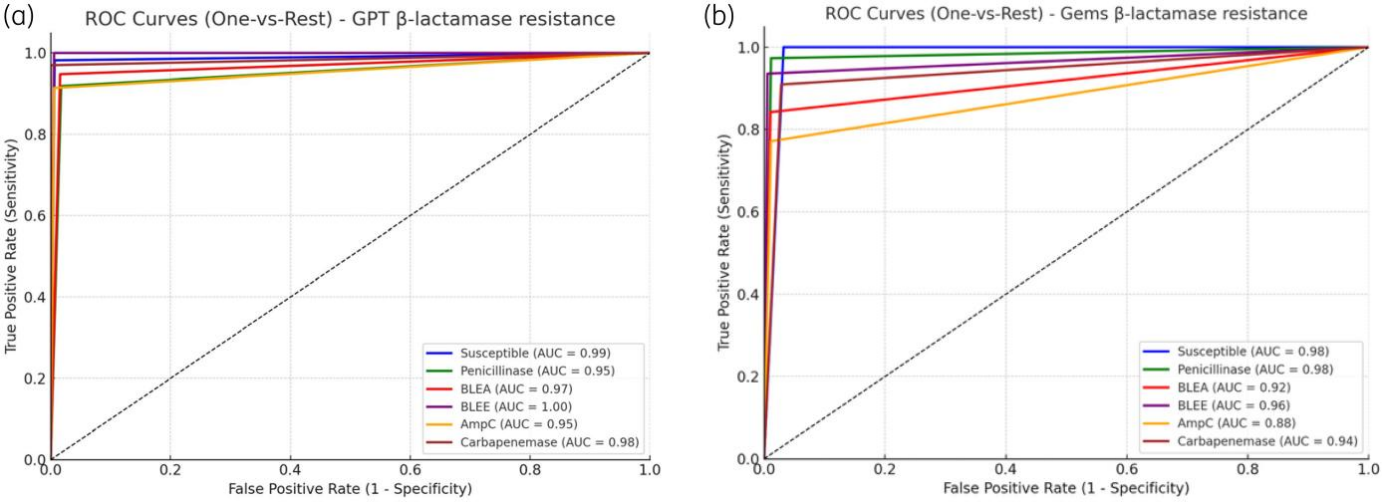
ROC curves of the models for the classification of resistance mechanisms (one-versus-rest): (a) GPT and (b) Gems.

**Figure 3.**
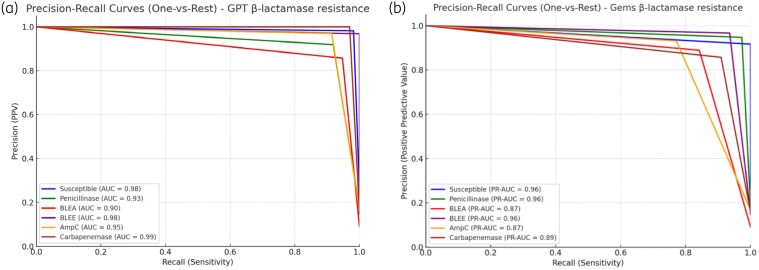
PR curves of the different models: (a) GPT and (b) Gems.

The ROC curves (Figure 2) demonstrated high discriminative ability for both models (AUC > 0.90), although GPT showed more uniform performance, with values approaching 1.0 for ESBL and Carba-R categories and exceeding 0.95 in all others. Gems also performed well but exhibited noticeable declines in BSBL and AmpC (AUC > 0.85). The PR curves (Figure 3) confirmed this pattern, highlighting that GPT maintains greater robustness and homogeneity across classes.

The error analysis (Table 2) showed that GPT made fewer clinically relevant errors (9) compared to Gems (17), with lower Spectrum Penalty Index (6.0 versus 13.0) and Failure Risk Index (1.15 versus 3.1). While GPT errors were mostly of low clinical impact, Gems exhibited a higher number of both overtreatment and undertreatment cases, indicating that GPT provides a safer performance with less potential clinical repercussion (see Supplementary material for a detailed explanation of this analysis).

**Table 2.** Comparison of clinically relevant error rates and associated impact indices between GPT and Gems

Indicator	GPT	Gems
Total errors	9.0	17.0
Overtreatment (*n*)	4.0	7.0
Undertreatment (*n*)	5.0	10.0
Spectrum Penalty Index	6.0	13.0
Failure Risk Index	1.15	3.1

## Discussion

One of the earliest attempts to apply AI in medicine was the development of systems to assist in antibiotic selection, notably MYCIN, a rule-based expert system created in 1973 that provided antibiotic recommendations with clinically acceptable performance for its time.^16^ However, the increasing complexity of AMR, with multiple emerging mechanisms and significant epidemiological variability, has made therapeutic decision-making increasingly challenging.^17^ In response, modern tools have evolved towards machine learning (ML) and generative systems capable of integrating phenotypic and molecular data, thereby improving the accuracy, speed and safety of clinical decisions related to rational antibiotic use.^18^

Several studies have shown that ML algorithms can enhance the prediction of AMR and assist clinicians in selecting appropriate empirical therapy. Techniques such as logistic regression, support vector machines, decision trees, random forest, XGBoost and deep neural networks have demonstrated promising performance, with AUC values ranging between 0.70 and 0.93.^19,20^ In parallel, ML-assisted AST methods have significantly reduced the time required for susceptibility profiling. For instance, the FASTinov system delivers results in under 2 h with >95% sensitivity and specificity;^21^ impedance-based assays can detect phenotypic changes within 30 min^22^ and infrared spectroscopy allows susceptibility estimation in approximately 40 min.^23^ Nevertheless, the widespread clinical adoption of these methods remains limited due to the need for specialized infrastructure, costly equipment and highly trained personnel.

In recent years, AI-based clinical decision-support systems (CDSS) integrating resistance predictions with local guidelines have achieved high precision in optimizing antimicrobial prescribing. For example, the Arkstone system, which combines molecular data with institutional guidelines, achieved 100% accuracy (*F*1 = 1) across 111 test cases,^24^ while another CDSS leveraging MALDI-TOF MS and random forest algorithms predicted fluoroquinolone resistance in *Klebsiella pneumoniae* with an AUC ≈ 0.95.^25^ These findings confirm the potential of ML models to improve empirical antibiotic selection tailored to local epidemiology.

LLMs have recently demonstrated remarkable potential in the interpretation of laboratory data, including antibiograms. When integrated with RAG, LLMs achieved a precision of 0.995 and an *F*1-score of 0.948, outperforming models without external data augmentation.^26^ In antimicrobial resistance prediction, LLM-based approaches (encoder-based models such as DNABERT2 and Nucleotide Transformer) have been applied to AMR gene classification from nucleotide sequences, with improved performance when sequence models are combined with external annotations such as BLASTn-derived information.^27^ These advances highlight the ability of LLMs to improve the speed, precision and personalization of diagnostic interpretation, supporting rational antimicrobial therapy.

A recent study further demonstrated the potential of generative models in diagnostic microbiology: the EUCAST-GPT-expert, a customized GPT-4 agent integrated with EUCAST guidelines and interpretative rules, successfully analysed disk diffusion images and classified isolates into ESBL, AmpC and Carba-R categories with sensitivities comparable to those of human microbiologists (95.4%, 96.9% and 100%, respectively), albeit with lower specificity and a tendency towards over-diagnosis requiring confirmatory testing.^28^ Unlike the non-specialized GPT-4, which could interpret only 19.6% of cases, the tailored agent achieved performance close to that of clinical experts and generated detailed reasoning outputs that may prove useful in educational and decision-support contexts. Nonetheless, further refinement and regulatory validation are needed before routine clinical implementation. Unlike our work, EUCAST-GPT-expert was designed mainly as a screening tool for flagging suspected resistance mechanisms from disk diffusion interpretation, a preliminary step that was not included in our workflow.

This study presents several strengths. The methodological design followed the STARD-AI guideline and incorporated a comprehensive statistical analysis, including accuracy metrics, ROC/PR curves and inter-model concordance. A key innovation was the evaluation of clinical impact, quantifying errors in terms of overtreatment and undertreatment through novel indices. Moreover, the direct comparison between two AI models provided valuable context to assess diagnostic safety and robustness. However, certain limitations must be acknowledged: convenience sampling and the relatively small size of some subgroups may restrict generalizability; the focus on Gram-negative bacilli and β-lactam resistance mechanisms excludes other clinically relevant pathogens; and the use of a single expert as the reference standard precluded assessment of inter-observer variability and may have introduced uncertainty regarding the reproducibility of the gold standard.

Although cases were selected to represent a single predominant resistance mechanism whenever possible, this approach may limit interpretation in complex isolates with combined mechanisms, ESBL or carbapenem-resistance heterogeneity, or inducible AmpC. In such scenarios, the generated report may appropriately refer to these possibilities rather than assign a single definitive mechanism; however, this behaviour was not specifically explored in the present study. This study focused on diagnostic accuracy under controlled conditions and did not assess workflow integration, user roles expert review procedures for hallucinations, omissions or commissions, or the impact of the system on routine laboratory practice.

Although promising, these findings should be considered proof-of-concept evidence rather than validation for immediate clinical deployment. Real-world implementation would require prospective multicentre validation, integration with laboratory and electronic health record systems, expert oversight, regulatory and data-governance assessment, continuous monitoring of clinically relevant errors and periodic updating according to evolving resistance patterns and interpretive guidelines. In addition, the model interprets completed antibiogram reports; it does not reduce the time required to perform AST, does not replace confirmatory or molecular testing, and was not evaluated in slow-growing organisms. In any future clinical implementation, the system should be framed strictly as a decision-support tool, with final diagnostic interpretation and therapeutic decisions remaining under the responsibility of qualified clinical microbiology, infectious disease and treating medical teams.

### Conclusions

This study provides proof-of-concept evidence that a custom GPT model can accurately interpret antibiograms of Gram-negative bacteria under controlled retrospective conditions, achieving high diagnostic accuracy and near-perfect concordance with expert standards. These findings support the feasibility of personalized generative AI models as potential decision-support tools in clinical microbiology. However, before real-world clinical deployment, prospective multicentre validation, workflow integration studies, regulatory assessment and continuous performance monitoring will be required to confirm generalizability, safety and clinical utility in routine practice.

## Supplementary Material

dlag132_Supplementary_Data
